# Supplementary material for: Large elasto-optic effect and reversible electrochromism in multiferroic BiFeO3
Source: Nat Commun. 2016 Feb 29;7:10718. doi: 10.1038/ncomms10718 (PMC4773452; doi:10.1038/ncomms10718)
Supplement: Supplementary Information — Supplementary Figures 1-3, Supplementary Table 1, Supplementary Note 1 and Supplementary References [file ncomms10718-s1.pdf]

Supplementary Figures

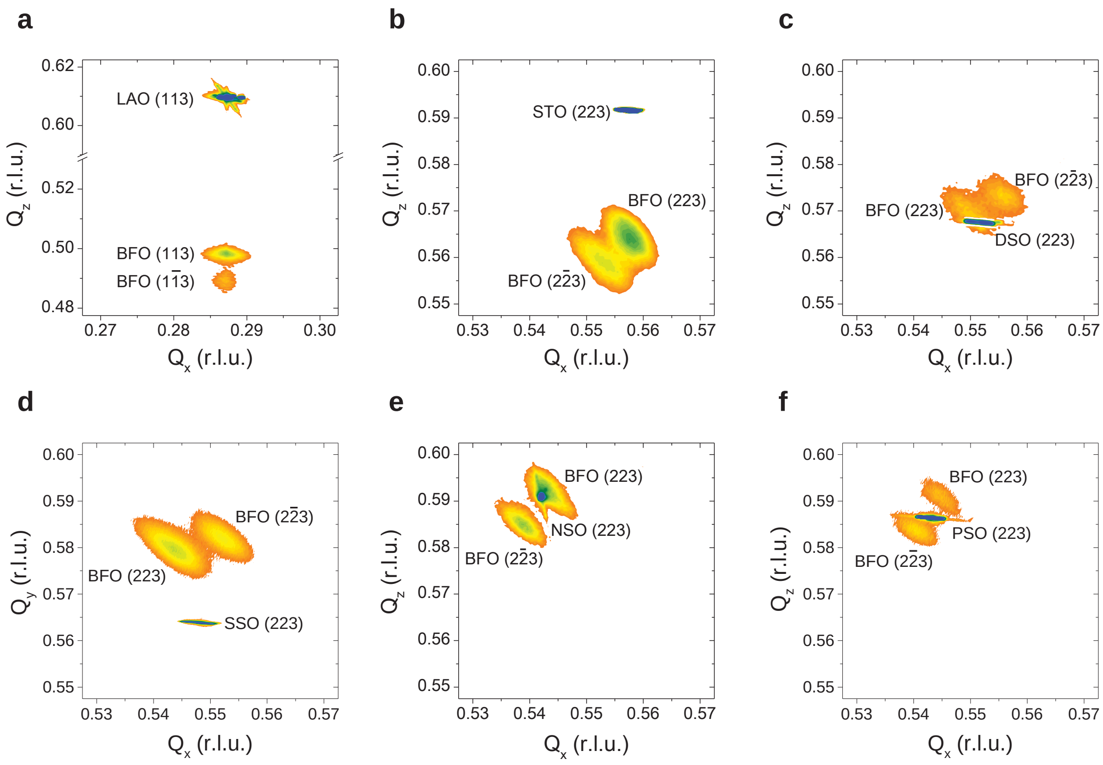

Supplementary Figure 1 | Reciprocal space mapping of $BiFeO_3$ films. To ascertain the monoclinic symmetry and nature of the twinning domains in our films, x-ray diffraction reciprocal space mapping was performed around the (113) (T-like) and (113) (R-like) reflections. (a) The T-like film on $LaAlO_3$ (LAO) shows an M_C monoclinic symmetry[1] with two structural variants, (b-c) the R-like films under compressive strain ($SrTiO_3$ – STO, $DyScO_3$ – DSO) were found to possess an M_A monoclinic structure[2] with two structural variants, and (d-e) the R-like films under tensile strain ($SmScO_3$ – SSO, $NdScO_3$ – NSO, $PrScO_3$ – PSO) have an M_B monoclinic symmetry[3] with two structural variants.

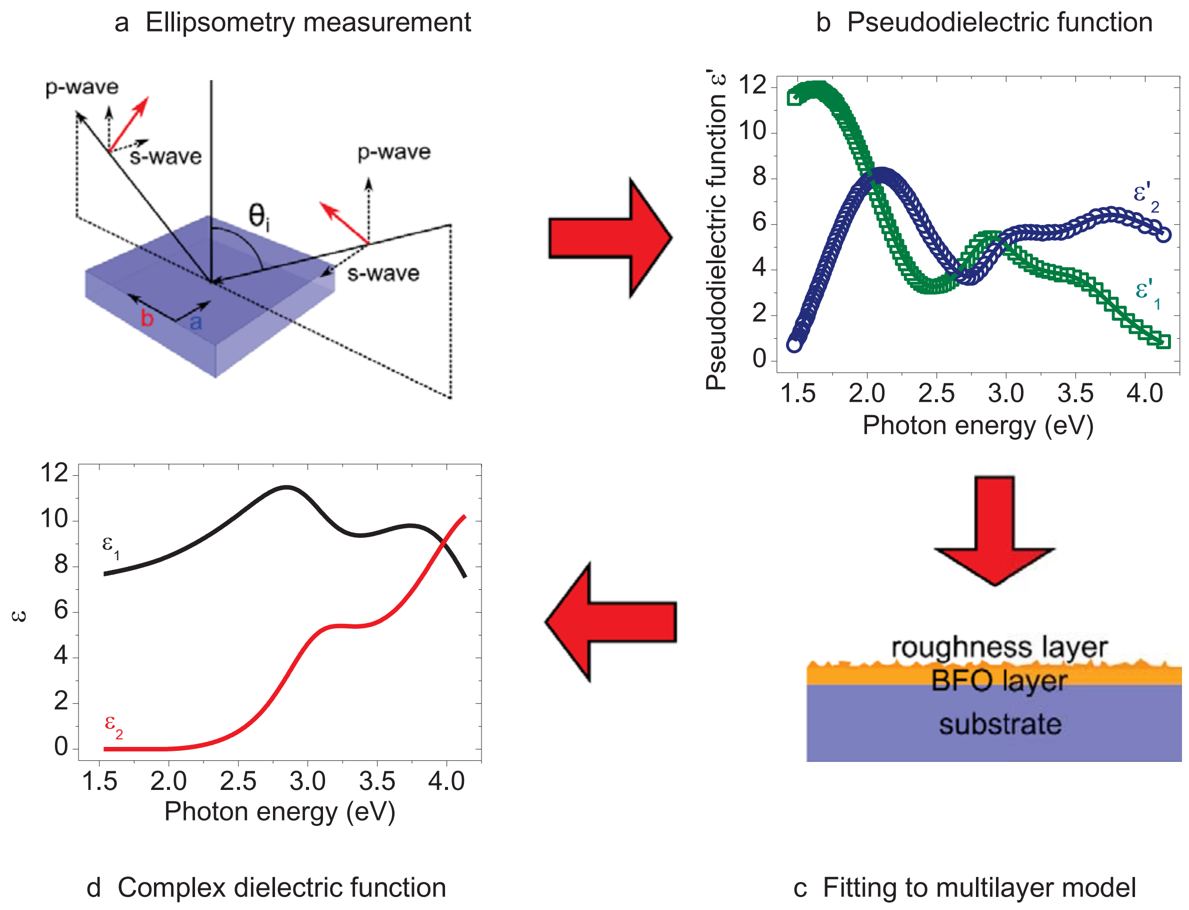

Supplementary Figure 2 | The process of spectroscopic ellipsometry as a flow chart. Spectroscopic ellipsometry[4] is a routine technique for the optical characterization of thin films. The complex reflection ratio (comprising the change in amplitude tan (Ψ) and phase Δ of the beam after reflection from the surface) yields information on the dielectric properties of the sample. To extract the optical constants, the data are acquired by the ellipsometer in the measurement geometry shown in (a), yielding the complex pseudodielectric function (b). Next, a multilayer model (c) is formulated incorporating the substrate, $BiFeO_3$ (BFO) layer and roughness layer, and fitting of the data with this model yields the complex dielectric function ($\tilde{\epsilon} = \epsilon_1 + i\epsilon_2$) of the film (d). BFO has a uniaxial optical anisotropy; however, since our films are mixed-domain, we consider only the average optical dielectric function. A typical fit of the data (for $BiFeO_3$//$NdScO_3$) is shown in (b); the data are shown as symbols, and the fits as lines.

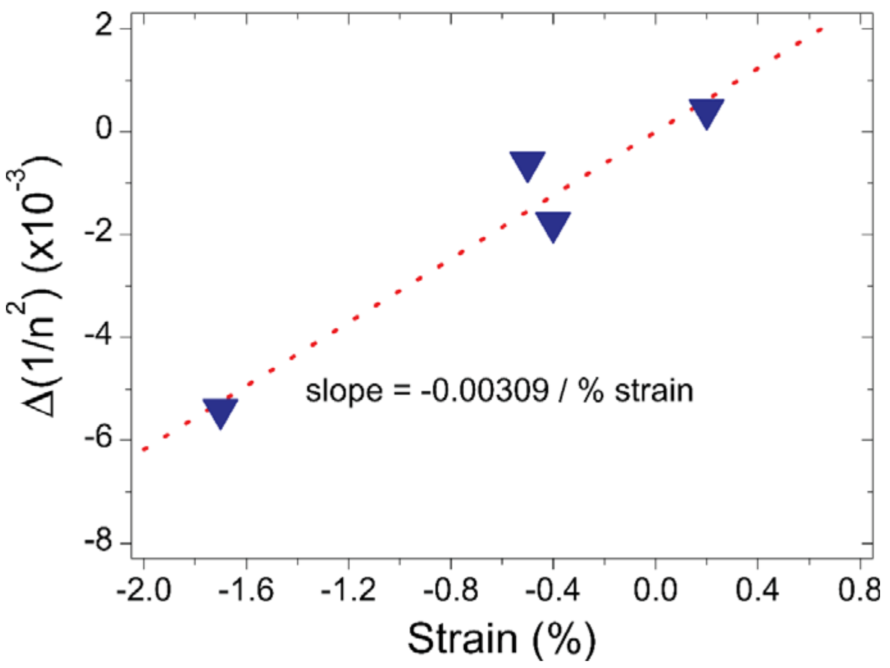

Supplementary Figure 3 | Calculation of effective elasto-optic coefficient at 500 nm.

Supplementary Table 1

SAMPLE				Additional Parameters				TL Oscillator 1						TL Oscillator 2						TL Oscillator 3					
		Av. $\chi 2$	Obs.	ε_∞		E_g		A_1		E_1		C_1		A_2		E_2		C_2		A_3		E_3		C_3	
Substrate	Strain (%)			value	±	value	±	value	±	value	±	value	±	value	±	value	±	value	±	value	±	value	±	value	±
LSAT	-2.6	0.10	4	1.5	0.1	1.99	0.01	28.5	0.1	3.047	0.004	1.000	0.005	33.8	0.2	4.168	0.005	1.435	0.007	33.4	0.8	5.70	0.03	0.350	0.002
STO	-1.6	0.15	8	2.18	0.04	1.95	0.02	30.1	0.2	3.03	0.02	0.960	0.005	15	1	4.00	0.02	0.920	0.005	39.8	0.2	5.09	0.08	1.2	0.3
DSO	-0.5	1.56	8	3.2	0.1	1.91	0.01	27.0	0.1	3.013	0.005	0.84	0.02	19.0	0.1	3.97	0.02	0.990	0.005	26.8	0.1	4.73	0.04	0.940	0.005
TSO	-0.4	0.25	4	2.8	0.1	1.80	0.01	23.0	0.1	3.035	0.006	0.85	0.01	20.8	0.1	3.99	0.02	0.98	0.01	22.5	0.1	4.77	0.07	0.750	0.004
SSO	+0.2	0.53	6	3.62	0.08	2.01	0.01	36.2	0.2	3.01	0.01	0.96	0.03	12.35	0.06	3.974	0.009	0.81	0.04	32.6	0.2	4.75	0.02	1.200	0.006
NSO	+0.9	0.40	8	2.83	0.04	1.76	0.01	17.3	0.1	3.062	0.005	0.75	0.02	26.2	0.1	4.07	0.03	1.130	0.006	19.2	0.1	4.80	0.09	0.450	0.002
PSO	+1.0	0.13	8	3.1	0.1	1.93	0.03	24.2	0.1	3.051	0.003	0.830	0.004	39.9	0.2	4.22	0.03	1.27	0.02	9.27	0.05	5.00	0.03	0.350	0.002
YAO	-7.0	0.10	4	2.58	0.05	1.63	0.03	12.59	0.06	3.30	0.01	1.3	0.2	14.20	0.07	4.21	0.03	1.53	0.05	22.3	0.1	5.44	0.03	1.570	0.008
LAO	-4.7	0.13	4	3.03	0.06	1.81	0.02	13.3	0.4	3.310	0.005	0.940	0.005	17.44	0.09	4.13	0.01	1.330	0.007	24.7	0.1	5.12	0.03	0.830	0.004

Supplementary Table 1 | Details of the Tauc-Lorentz (TL) parameters for the ellipsometry fits. Obs. = number of measurements on sample; Av. χ^2 = average mean-square error of fits for a given sample. Each TL oscillator is defined by an energy E_0, amplitude A and broadening factor C. In addition, for each model there are two other fitting parameters, ε_∞ and E_g.

Supplementary Note 1 | Calculation of effective elasto-optic coefficient

Here, we considered the weakly-compressively-strained samples (the films grown on STO, TSO, DSO, and SSO substrates, which impart -1.7%, -0.5%, -0.4% and +0.2% strain respectively). The elasto-optic effect describes the change in refractive index of a medium induced by a deformation through[5]

$$\Delta\left(\frac{1}{n^2}\right)_{ij} = \sum_{k,l} p_{ijkl}\, \varepsilon_{kl},$$

where p is the tensor of elasto-optic coefficients, and ε is the strain tensor (note that strain here is defined as absolute, not in %). Since our films are mixed domain, we cannot isolate a specific tensor element, so we consider the average, or effective elasto-optic coefficient p_{eff} which can be described by the simplified relation

$$\Delta\left(\frac{1}{n^2}\right) = p_{\mathrm{eff}} \times \varepsilon.$$

The unstrained value of refractive index n_0 is determined as the intercept of a linear fit to the n data in Fig. 4b. Then $\Delta(1/n^2)$ is calculated as the difference between n_0 and the refractive index measured at a particular strain. These values form a linear trend and as shown in Fig. S3, the slope of the linear regression line yields the value of p_{eff}. Performing this process for the refractive index data at various wavelengths allowed us to construct Fig. 4d.

Supplementary References

1. Chen, Z. *et al.* Low symmetry monoclinic M_C phase in epitaxial $BiFeO_3$ thin films on $LaSrAlO_4$ substrates. *Appl. Phys. Lett.* **97,** 242903 (2010).

2. Xu, G. *et al.* Low symmetry phase in (001) $BiFeO_3$ epitaxial constrained thin films. *Appl. Phys. Lett.* **86,** 182905 (2005).

3. Chen, Z. *et al.* Large tensile-strain-induced monoclinic M_B phase in $BiFeO_3$ epitaxial thin films on a $PrScO_3$ substrate. *Phys. Rev. B* **88,** 054114 (2013).

4. Tompkins, H. & Irene, E. *Handbook of ellipsometry*. (William Andrew, 2005).

5. Dieulesaint, E. & Royer, D. *Elastic Waves in Solids II*. (Springer-Verlag Berlin Heidelberg, 2000).
